# Supplementary material for: Resolving the full spectrum of human genome variation using Linked-Reads
Source: Genome Res. 2019 Apr;29(4):635–45. doi: 10.1101/gr.234443.118 (PMC6442396; doi:10.1101/gr.234443.118)
Supplement: Supplemental Material [file supp_gr.234443.118_Supplemental_longranger-2.2.zip › longranger-2.2.tar.bz2!/lr2.2-public/lib/go/src/code.google.com/vitess/docs/getting-started/docker-build.html]

Vitess / Custom Docker Build


Toggle navigation

- Overview
- Guides
- Reference
- Blog
- GitHub

- #### Overview

  - What is Vitess
  - Scaling MySQL with Vitess
  - Key Concepts
- #### Getting Started

  - Run Vitess on Kubernetes
    - Custom Docker Build
  - Run Vitess Locally
- #### User Guide

  - Introduction- Client Libraries- Backing Up Data- Reparenting
        - Schema Management
        - VSchema Guide
        - Schema Swap (Tutorial)
        - Sharding
          - Horizontal Sharding (Tutorial, automated)
          - Horizontal Sharding (Tutorial, manual)
          - Sharding in Kubernetes (Tutorial, automated)
          - Sharding in Kubernetes (Tutorial, manual)
        - Vitess Sequences
        - MySQL Server Protocol
        - Vitess and Replication
        - Update Stream
        - Row Based Replication
        - Topology Service
        - Transport Security Model
        - Launching
          - Scalability Philosophy
          - Production Planning
          - Server Configuration
          - 2PC Guide
          - Troubleshooting
        - Upgrading
- #### Reference Guides

  - Vitess API- vtctl Commands
- #### Other Resources

  - Presentations- Blog- Roadmap
- #### Contributing

  - Contributing to Vitess- GitHub Workflow- Code Reviews
- GitHub

# Custom Docker Build

- #### Overview

  - What is Vitess
  - Scaling MySQL with Vitess
  - Key Concepts
- #### Getting Started

  - Run Vitess on Kubernetes
    - Custom Docker Build
  - Run Vitess Locally
- #### User Guide

  - Introduction- Client Libraries- Backing Up Data- Reparenting
        - Schema Management
        - VSchema Guide
        - Schema Swap (Tutorial)
        - Sharding
          - Horizontal Sharding (Tutorial, automated)
          - Horizontal Sharding (Tutorial, manual)
          - Sharding in Kubernetes (Tutorial, automated)
          - Sharding in Kubernetes (Tutorial, manual)
        - Vitess Sequences
        - MySQL Server Protocol
        - Vitess and Replication
        - Update Stream
        - Row Based Replication
        - Topology Service
        - Transport Security Model
        - Launching
          - Scalability Philosophy
          - Production Planning
          - Server Configuration
          - 2PC Guide
          - Troubleshooting
        - Upgrading
- #### Reference Guides

  - Vitess API- vtctl Commands
- #### Other Resources

  - Presentations- Blog- Roadmap
- #### Contributing

  - Contributing to Vitess- GitHub Workflow- Code Reviews

By default, the Kubernetes configs
point to the `vitess/lite` image on Docker Hub.
This image is built periodically from the master branch on GitHub.

If you want to customize this image, you can build your own like this:

1. Install Docker on your workstation.

   Our scripts also assume you can run the `docker` command without `sudo`,
   which you can do by setting up a docker group.
2. Create an account on Docker Hub and
   then `docker login` to it.
3. Go to your `github.com/youtube/vitess` directory.
4. Usually, you won't need to build your own bootstrap image
   unless you edit bootstrap.sh
   or vendor.json,
   for example to add new dependencies. If you do need it then build the
   bootstrap image, otherwise pull the image using one of the following
   commands depending on the MySQL flavor you want:

   ```
   vitess$ docker pull vitess/bootstrap:mysql57   # MySQL Community Edition 5.7
   vitess$ docker pull vitess/bootstrap:mysql56   # MySQL Community Edition 5.6
   vitess$ docker pull vitess/bootstrap:percona57 # Percona Server 5.7
   vitess$ docker pull vitess/bootstrap:percona   # Percona Server
   vitess$ docker pull vitess/bootstrap:mariadb   # MariaDB
   ```

   **Note:** If you have already downloaded the `vitess/bootstrap:<flavor>`
   image on your machine before then it could be old, which may cause build
   failures. So it would be a good idea to always execute this step.
5. Build the `vitess/lite[:<flavor>]` image. This will build
   `vitess/base[:<flavor>]` image and run a script that extracts only the files
   needed to run Vitess (`vitess/base` contains everything needed for
   development work). You will be asked to authenticate with `sudo`, which is
   needed to fix up some file permissions.

   Choose one of the following commands (the command without suffix builds
   default image containing MySQL 5.7):

   ```
   vitess$ make docker_lite
   vitess$ make docker_lite_mysql56
   vitess$ make docker_lite_percona57
   vitess$ make docker_lite_percona
   vitess$ make docker_lite_mariadb
   ```
6. Re-tag the image under your personal repository, then upload it.

   ```
   vitess$ docker tag -f vitess/lite yourname/vitess
   vitess$ docker push yourname/vitess
   ```

   **Note:** If you chose non-default flavor above then change `vitess/lite` in
   the above command to `vitess/lite:<flavor>`.
7. Change the Kubernetes configs to point to your personal repository:

   ```
   vitess/examples/kubernetes$ sed -i -e 's,image: vitess/lite,image: yourname/vitess:latest,' *.yaml
   ```

   Adding the `:latest` label at the end of the image name tells Kubernetes
   to check for a newer image every time a pod is launched.
   When you push a new version of your image, any new pods will use it
   automatically without you having to clear the Kubernetes image cache.

   Once you've stabilized your image, you'll probably want to replace `:latest`
   with a specific label that you change each time you make a new build,
   so you can control when pods update.
8. Launch Vitess on Kubernetes as usual.

Contact: vitess@googlegroups.com  **·**  
Announcements  **·**  
© 2017 Vitess powered by Google Inc
